# One Size Fits None: Developing a Person‐Centered Approach to Weight Management in Older Adults

**DOI:** 10.1111/jgs.70586

**Published:** 2026-07-11

**Authors:** Aruna V. Josyula, Jennifer Muniak, Anushka Tandon, Noelle Marie Javier, Michi Yukawa, Colleen Christmas, Timothy W. Farrell

**Affiliations:** ^1^ Central Texas Veterans Health Care System Temple Texas USA; ^2^ Texas A&M University, Vashisht College of Medicine College Station Texas USA; ^3^ University of Rochester Rochester New York USA; ^4^ U.S. Department of Veterans Affairs Edward Hines Jr. VA Hospital Hines Illinois USA; ^5^ Brookdale Department of Geriatrics and Palliative Medicine Icahn School of Medicine at Mount Sinai New York New York USA; ^6^ Department of Medicine, Division of Geriatrics University of California San Francisco San Francisco California USA; ^7^ Short Stay Rehabilitation Unit San Francisco Campus for Jewish Living San Francisco California USA; ^8^ Johns Hopkins School of Medicine Baltimore Maryland USA; ^9^ Division of Geriatrics, Department of Internal Medicine Spencer Fox Eccles School of Medicine, University of Utah Salt Lake City Utah USA; ^10^ VA Salt Lake City Geriatric Research, Education, and Clinical Center Salt Lake City Utah USA

**Keywords:** anti‐obesity medications, older adults, sarcopenia, weight management, weight stigma

## Abstract

- Key Points
  ○ Weight management in older adults requires an understanding of age‐related physiologic and psychosocial differences relative to a younger adult population.
  ○ Cultural humility and trauma‐informed language guide safe and respectful discussions about weight.
  ○ Effective communication regarding weight management incorporates the Geriatrics 5Ms framework, patient permission, and stigma reduction strategies.
  ○ Intentional weight loss should prioritize preservation of lean mass and functional safeguards.
- Why Does This Paper Matter?
  ○ Weight management interventions in older adults carry unique clinical risks and treatment discussions are often complicated by stigma.
  ○ This commentary provides a practical, person‐centered framework to help clinicians conduct safe, empathetic weight‐related conversations aligned with What Matters Most to patients.

## 1 Background

Clinicians are navigating a rapidly evolving weight management landscape driven by the classification of obesity as a chronic disease and recent pharmacologic advances. While BMI (Body Mass Index) is the most commonly assessed clinical measure to gauge weight management, it does not account for the way sex, gender, race, ethnicity, body composition, or other individual characteristics may impact treatment decisions. Therapeutic modalities for intentional weight loss include lifestyle modifications (diet and exercise), bariatric surgery, and anti‐obesity medications (AOMs), such as glucagon‐like peptide‐1 receptor agonists (GLP‐1RAs) like semaglutide and dual receptor agonists (glucose‐dependent insulinotropic polypeptide [GIP]/GLP‐1RAs) like tirzepatide. Age‐related changes in pharmacokinetics, pharmacodynamics, multimorbidity, and polypharmacy increase older adults' risk of experiencing adverse effects from weight management interventions. Ongoing prescribing expansion and copay reduction efforts continue to increase AOM accessibility for patients. Clinicians can expect to address the topic of weight management more frequently with older adults due to increased patient interest in intentional weight loss or as part of comprehensive care planning efforts to address comorbid conditions. This commentary addresses how to apply the Geriatrics 5Ms framework (What Matters Most, Mind, Medications, Mobility, and Multicomplexity) to engage older adults in respectful, evidence‐based, person‐centered discussions about weight (Figure 1) [1].

**FIGURE 1:**
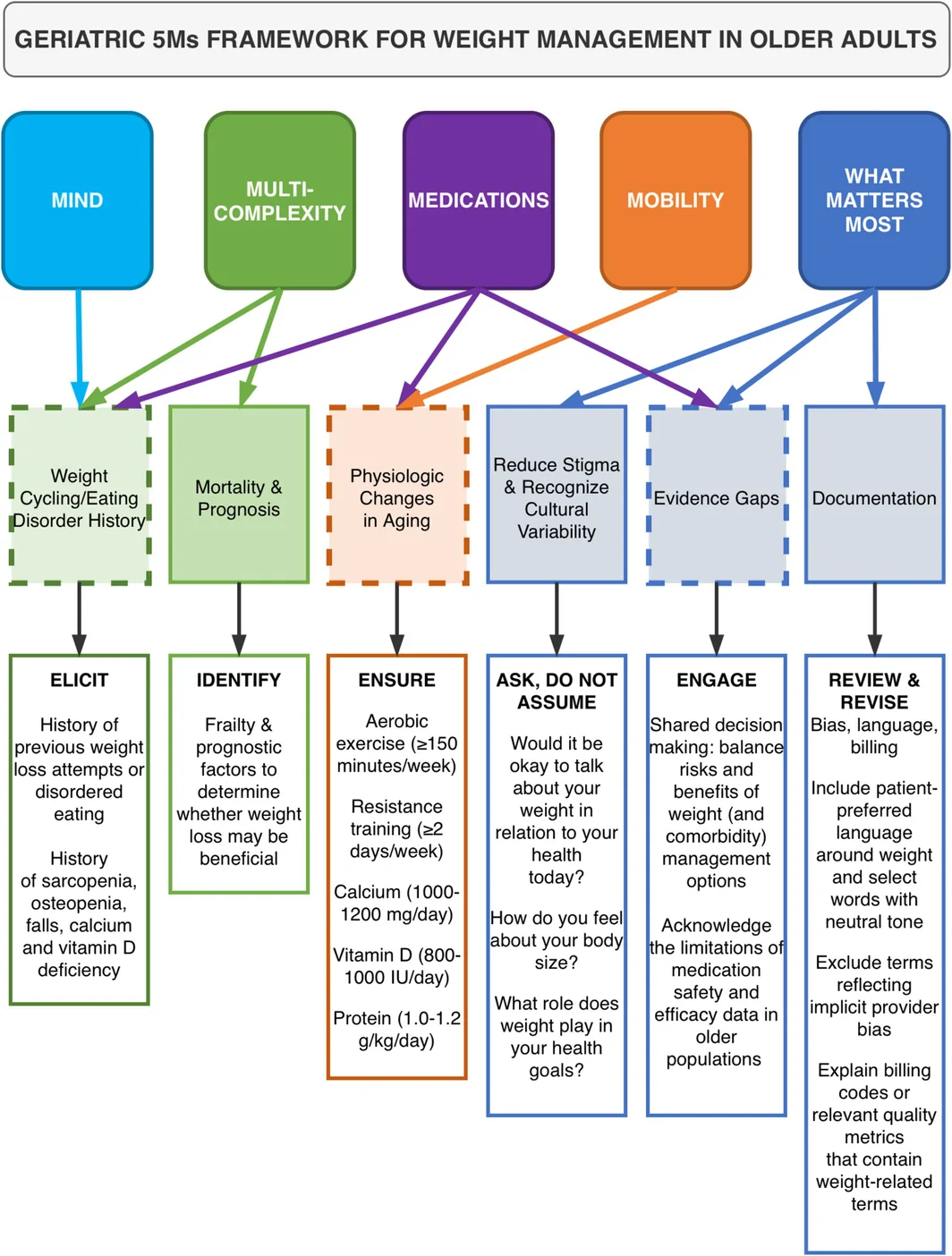
Colored arrows and boxes = 5 M domain connections Gray arrows = Flow to practical approaches for implementation Dashed boxes = Shared considerations (multiple 5 M domain connections). This figure was created with assistance from OpenEvidence, which was used to convert Figure 1's outline of content and structure into XML code for use with the draw.io diagramming tool.

### 1.1 Person‐Centered Considerations: Rethinking Weight Narratives (What Matters Most)

#### 1.1.1 Reducing Stigma

Weight stigma, or the misconceptions and stereotypes associated with higher body weight, is an important social determinant of health. The pervasive societal stereotyping of individuals with higher body weights as lazy, gluttonous, or unintelligent also extends to healthcare settings. Stigmatization can lead to medical discrimination of patients, resulting in care avoidance and delays, psychological distress, and worse overall health outcomes. Clinicians may inadvertently perpetuate weight stigma and patient discrimination by focusing recommendations around managing weight rather than improving health. Using trauma‐informed and responsive techniques to guide weight‐related conversations can help clinicians address patients with respect and humility while mitigating communicated bias and discrimination; this can help foster curiosity, open‐mindedness, and trust within therapeutic relationships. Techniques to consider include the following: [2]

- Ask for permission to broach weight‐related discussions
- Refrain from stating assumptions about patients' intended goals
- Solicit patient choice regarding non‐stigmatizing terminology to use (e.g., fat, obese, larger body, higher body weight)
- Employ a neutral tone (e.g., “adiposity‐related chronic disease”)
- Utilize person‐first language (“patient with obesity” rather than “obese patient”)
- Abstain from assigning personal or moral responsibility around weight changes

Beyond individual or interpersonal considerations, several systemic factors also contribute to perpetuating weight stigma. These include, but are not limited to [2]:

- Pejorative standardized language embedded in electronic health records (EHR)
- Performance on weight‐centric quality measures as an indicator of clinical excellence
- Weight discrimination in hiring practices
- Unaccommodating environments and design
- Public health messaging around obesity as an “epidemic”
- Popular media and wellness culture portrayals of higher body weights

Recommendations on how to address these systemic factors through policy‐driven or legislative approaches are beyond the scope of this clinician‐focused commentary. However, we recognize that it is essential to consider the intersection of weight stigma with structural racism and ageism [3] when developing systemic strategies for harm reduction in older adult care.

#### 1.1.2 Understanding Factors Influencing Perceptions and Disparities Around Weight Management

Both clinician‐level and patient‐level characteristics may impact the quality of weight management‐related care.

In a United States survey [4], physicians with normal BMI (defined as self‐reported BMI < 25 kg/m^2^) reported greater confidence in providing diet and exercise counseling to patients with obesity than physicians with higher BMI values. They also felt that patients would be less likely to trust weight management advice provided by clinicians with higher body weights.

Cultural norms are widely understood to shape perceptions of body size and weight. While some cultures idealize thinness, others are more accepting of larger body sizes; religious beliefs may also influence weight‐related attitudes. Individuals may experience conflict between the cultural perception of their higher weights as “healthy” and their personal interests in health‐motivated weight loss. Additionally, socioeconomic factors can impact access to and affordability of healthy food, physical activity, and weight management interventions. These variables are significant determinants of the disparities in obesity prevalence, diagnosis, and management across racial and ethnic minority populations [5]. Black and Hispanic adults have significantly higher odds of obesity (OR 1.97 and 1.81) and obesity‐related conditions, including diabetes (OR 2.58 and 2.71), hypertension, stroke, and heart attack than White adults [6].

### 1.2 Clinical Considerations: Older Adults are not Younger Adults

#### 1.2.1 Morbidity, Mortality, and Prognosis (Multicomplexity)

Aging alters the risk–benefit calculus for weight loss. The BMI‐mortality relationship in older adults follows a J‐shaped curve (Figure 2) [7], with the lowest risk of death in the overweight (25.0–29.9 kg/m^2^) to Class 1 obesity (30.0–34.9 kg/m^2^) range. Aggressive weight loss is rarely appropriate for those with advanced illness, extreme frailty, or short life expectancy. These individuals may already be at risk for unintentional weight loss from underlying conditions, depression, dentition issues, or cognitive impairment [8]. Further caloric restriction in this context can accelerate malnutrition and worsen prognosis.

**FIGURE 2:**
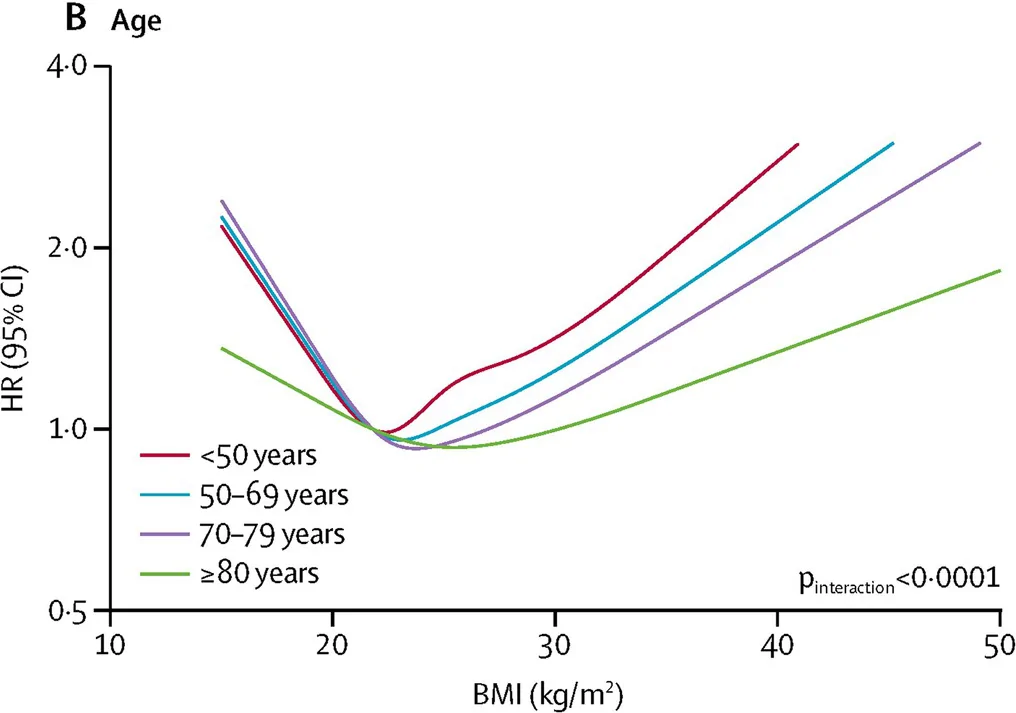
Reproduced without changes from: Association of BMI with overall and cause‐specific mortality: A population‐based cohort study of 3.6 million adults in the UK. The Lancet [7]. Licensed under CC BY 4.0 (https://creativecommons.org/licenses/by/4.0/).

#### 1.2.2 Physiologic Changes in Aging (Mobility, Medications)

Age‐related fat redistribution and musculoskeletal loss predispose older adults to obesity, sarcopenia, and osteoporosis [9]; rapid or profound weight reduction can accelerate these processes. Approximately 25% of weight lost with GLP‐1 therapies is lean (bone and muscle) mass [10]. Bone mineral density (BMD) changes with these medications are mixed—declines appear driven by weight loss in non‐diabetic populations, while BMD is neutral or improved in patients with diabetes [11, 12, 13]. BMD declines are not consistently linked to increased fracture incidence, though a recent database case–control study suggests significantly increased GLP‐1‐associated fracture risk in nondiabetic patients with BMI ≥ 40 or age > 67 after 1 year [14]. Patient age, sex, physical activity, nutrition, and rate of weight change can significantly influence lean mass loss. Therefore, gradual weight loss paired with structured nutrition and exercise support are advised to mitigate loss of fat‐free mass [15], though the degree to which adverse changes can be mitigated remains unclear. Sarcopenic obesity, associated with increased morbidity, mortality, and cognitive impairment risks, affects 11% of older adults globally [16]. As GLP‐1‐specific nutrition and exercise guidelines are not yet available, the following general recommendations are advised to minimize treatment‐associated risks for older adults [9, 17]:

- Multidisciplinary support
- Weight‐bearing aerobic exercise ≥ 150 min/week
- Resistance training ≥ 2 days/week
- Moderate caloric restriction ~500 kcal/day
- Adequate protein intake 1–1.2 g/kg/day
- Calcium intake 1000–1200 mg/day
- Vitamin D intake 800–1000 IU/day

#### 1.2.3 Weight Cycling and Eating Disorder History (Mind, Multicomplexity, Medications)

Beyond routine screening for causes of weight changes (e.g., endocrine disorders, cancer, HIV/AIDS, depression), clinicians should also assess weight cycling and eating disorders. Older adults with longstanding overweight or obesity may have a decades‐long history of weight loss attempts. After weight regain, older adults disproportionately replace lean muscle with fat compared to their younger counterparts, increasing sarcopenia risk [18]. Although evidence on prevalence is limited, case reports demonstrate that older adults can experience eating disorders with serious clinical consequences [19]. Disordered eating may coexist with overweight or obesity, complicating decisions about prescribing AOMs.

#### 1.2.4 Evidence Gaps (What Matters Most, Medications)

Adults aged ≥ 65, and especially ≥ 75, remain underrepresented in clinical trials, limiting generalizability of study outcomes. While GLP1‐RA trials for diabetes treatment enrolled a substantial older adult population, fewer than 10% of participants in obesity treatment trials were older adults [20]. Research regarding optimal pharmacotherapy duration and validated tapering strategies is also lacking; clinical trials showed that within 1 year of sudden treatment discontinuation, one‐half to two‐thirds of lost weight is regained and associated cardiometabolic benefits are reversed [11]. These gaps in evidence underscore the need for shared‐decision making.

### 1.3 Documentation: Words Matter (What Matters Most)

Electronic health record access has made clinical documentation visible to patients and their caregivers. Care notes may reveal implicit clinician biases, display stigmatizing language used in billing codes and quality indicators, and provoke strong emotional reactions in patients, all of which can impact the therapeutic relationship between patients and clinicians. To cultivate patient rapport and ensure delivery of respectful care, clinicians must be intentional in their efforts to adopt:

- Patient‐chosen terminology
- Neutral terms (e.g., adiposity‐related chronic disease or BMI rather than obesity)
- Person‐first phrasing

## 2 Conclusion

Older adults with higher body weight face pervasive cultural biases and structural barriers to optimal care. Navigating weight management in a person‐centered way requires skill, practice, creativity, resources, and advocacy. Clinicians should proceed thoughtfully, given the heightened risks of weight loss and weight cycling in older adults compared with younger populations. Importantly, they should avoid reliance on chronological age alone to determine treatment candidacy. Weight management strategies should be developed with consideration for targeted underlying conditions, prognosis, and What Matters Most to each individual patient.

## Author Contributions

All authors assisted with the conception and design of this commentary, reviewed and interpreted evidence used to inform recommendations, drafted and critically revised the manuscript for clarity of content, and approved the final version for submission. The authors wish to acknowledge the use of OpenEvidence AI to translate Figure 1's outline of content and structure into XML code for use with the draw.io diagramming tool.

## Conflicts of Interest

Dr. Yukawa receives royalties for authorship of the UpToDate chapter “Geriatric Nutrition: Nutritional Issues in Older Adults.” The other authors have no conflicts of interest to disclose.
